# Wealth inequalities in childhood stunting in the northern province of Rwanda: a decomposition analysis

**DOI:** 10.1186/s12939-025-02626-9

**Published:** 2025-09-29

**Authors:** Albert Ndagijimana, Miguel San Sebastian, Kristina Elfving, Aline Umubyeyi, Torbjörn Lind

**Affiliations:** 1 Department of Clinical Sciences, Umeå University, Paediatrics, Umeå, Sweden; 2 College of Medicine and Health Sciences, School of Public Health, University of Rwanda, Kigali, Rwanda; 3 Department of Epidemiology and Global Health, Umeå University, Umeå, Sweden; 4 School of Public Health and Community Medicine, Gothenburg University, Queen Silvia’s Children Hospital, Gothenburg, Sweden

**Keywords:** Concentration index, Decomposition, Inequalities, Rwanda, Socio-economic, Undernutrition

## Abstract

**Background:**

Childhood stunting remains a public health challenge, especially in sub-Saharan Africa. In Rwanda, it is the highest in the Northern province, with a prevalence of 40.5% in children under five. Given that poverty is a key determinant of stunting, we aimed to investigate wealth inequalities in childhood stunting and to identify its contributing determinants in the province.

**Methods:**

We included 601 children aged 1 to 36 months. We estimated the concentration index to quantify wealth-related inequalities in stunting, which we further decomposed it by applying the Wagstaff decomposition approach.

**Results:**

The overall concentration index of child stunting was relatively high (-0.190; 95% CI: -0.295, -0.084), suggesting that stunting was concentrated in poorer households. Its decomposition revealed that socio-economic determinants (147.9%), namely wealth index, partner’s unemployment or non-skilled occupation, and household food insecurity, contributed most to the wealth inequalities of childhood stunting. The demographic determinants contributed in the second order, especially living in Musanze district. Psychosocial determinants came last, namely maternal social support.

**Conclusion:**

This study revealed significant wealth inequalities in childhood stunting in the Northern Province of Rwanda, with socio-economic factors being the primary contributors to these inequalities. Targeted interventions, such as household economic strengthening programs, robust food security policies, improved conditions and salaries in non-skilled jobs, and social support programs for the most disadvantaged communities, are essential to reducing these disparities.

## Background

Undernutrition, which has an estimated worldwide prevalence of 22% among children aged under five years, remains a significant contributor to child mortality and disease burden [1]. Its chronic form, stunting, is the most prevalent, affecting 148.1 million (22.3%) children under five globally in 2022 [2]. Up to 64% of all stunted children live in low- and middle-income countries (LMICs), where sub-Saharan Africa has a prevalence of 31.3% and Eastern Africa has 21.8% [2]. In Rwanda, an estimated 33% of children aged 6–59 months are stunted, and its Northern Province has the highest prevalence, 40.5% according to the 2019–2020 Rwanda Demographic and Health Survey (RDHS) [3]. Stunting affects child development and cognitive function, which hinders the economic development of families, societies, and nations [4]. To reverse these effects, there is a need to invest in the first 1000 days of childhood to alleviate malnutrition [5].

The most common determinants of child malnutrition are well documented, and these mainly include poverty, preterm birth or small-for-gestational-age births, recurrent child infections exacerbated by poor sanitation and unsafe drinking water, food insecurity, limited access to health services, inadequate childcare and feeding practices, poor maternal health and nutrition, inadequate maternal education, short birth intervals, and large family size [6, 7].

In nearly all epidemiological studies on childhood stunting, poverty consistently emerges as a critical predictor [6]. Children from poor families are more likely to be stunted than children from affluent families due to exposure to pathogens, poor childcare practices, high likelihood of illness, and poor coverage of preventive interventions, including lower access to health services [8]. Given that poverty is the main driver, it is vital to understand the determinants of wealth inequalities in the population.

Some studies have attempted to examine socio-economic inequalities in stunting in Rwanda. One study compared determinants between rural and urban settings in Rwanda and Burundi, finding low socio-economic status at the household level to be associated with chronic malnutrition, but only in rural settings [9]. Another recent study with a meta-analysis of 25 studies estimated that 75% of the overall inequality in stunting was due to the difference in the social determinants between poor and non-poor households [10]. Using two rounds of DHS data, another study performed a rural-urban decomposition in stunting to find child’s age, number of antenatal care visits, and wealth as the main determinants of the reduction of moderate and severe stunting in Rwanda [11]. In addition to this evidence, further equity analysis with an emphasis on wealth as the primary contributor to inequalities in areas with higher prevalence, such as the Northern province, can inform more targeted actions by the government and partners in child health. By removing the wealth inequalities, the country would be able to contribute to the global target of reducing the number of stunted children by 40% points by 2025 [12]. In the same direction, the current and new health sector strategic plan states the ambition to reduce the prevalence of stunting among children under five to 15% by the year 2029, which entails reinforcing a multisectoral approach [13]. Until there is evidence on how disproportionately stunting is affecting children between wealth categories, key contributors, and the extent to which that disproportion occurs, it is hard to appreciate the relevance of interventions.

To address this knowledge gap, this study employs a complex equity analysis method, combined with a decomposition approach, to quantify wealth-related inequalities in child stunting in the Northern Province of Rwanda and identify the key socio-economic and demographic determinants contributing to these inequalities.

## Materials and methods

### Design, population and sampling

We conducted a population-based, cross-sectional study in the Northern Province of Rwanda between October-December 2021. The sample size was determined as:

$$\:n=\frac{{Z}_{\propto\:/2}^{2}\times\:\:\text{p}\:\times\:\:\left(1-p\right)}{{d}^{2}}\times\:DEFF\:$$

whereby n is the required sample size, Zα/2 is the critical value of the normal distribution at α/2 (e.g., for a confidence level of 95%, α is 0.05 and the critical value is 1.96), d is the degree of precision (0.05), p is the proportion of stunting in Northern Province (40.5%) [3], and DEFF is the design effect (1.5). A sample of 553 households with eligible children was estimated, which was increased to 615 households after applying a non-response rate of 10%.

We used a two-stage cluster sampling technique to select households. First, we randomly selected 137 enumeration areas (EAs) from the five districts of the province (Musanze, Burera, Gicumbi, Rulindo, and Gakenke). Second, we randomly selected 615 households with children aged 1–36 months. We obtained the sampling frame (updated list of households per village) from the community health workers in charge of maternal and child health at the village level. At the household level, we targeted the index child’s biological mother and collected data on child and maternal characteristics.

### Data collection methods

We used a structured questionnaire to collect data on socio-economic and demographic characteristics of the household (e.g., assets, education, and main occupation) and potential determinants of undernutrition. Child anthropometric measurements (length/height, weight, head circumference, and mid-upper arm circumference) were recorded using UNICEF-designed height charts, digital weight scales (SECA AG, Hamburg, Germany), and tape measures.

### Measures

#### Outcome variable: childhood stunting

To estimate stunting in children aged 1–36 months, our outcome variable, we used their anthropometric measurements (height in centimeters and age in months). We then calculated the height-for-age z-scores using the WHO Antro version 3.2.2, and categorized them according to the WHO standards for child growth [14]. Children were classified as stunted if the HAZ score values were more than 2 standard deviations (SD) below the median [15].

#### Socio-economic indicator: wealth

We estimated the household socio-economic status in terms of asset score as a proxy of socio-economic indicator (wealth) by using principal component analysis (PCA), a method that has always been used in demographic and health surveys [16]. Principal component analysis (PCA) was applied to variables related to assets, such as household possessions (mattress, radio, television, refrigerator, motorcycle, bicycle, iron, mobile phone), housing features (toilet type, water source, electricity, building materials), and land and livestock ownership. PCA transforms these potentially correlated variables into uncorrelated components that capture the variance in the data. The first principal component, explaining the largest share of variance, was used to generate a composite household wealth score. From this score, quintiles were created, ranging from the first (poorest) to the fifth (richest) group of households.

#### Inequality contributors

Variables were organized according to demographic, socio-economic, and psychosocial factors based on the WHO framework for stunting [17]. Data on key demographic characteristics such as the child’s age in months as nutrition-sensitive windows (categorized in 1–11, 12–23, and 24–36) and gender (categorized as male and female), the mother’s age in years (categorized as 17–29, 30–39, and 40–59) and marital status of the mother (categorized as single, cohabitant, and married), and the district of residence, were collected.

In terms of socio-economic variables, maternal education was categorized as less than primary, primary, secondary, or higher education, and recorded for both mothers and their partners. The main occupation of the mother’s partner was categorized as either unemployed or employed. Household food security was categorized as household food secure, mildly food insecure, moderately food insecure, or severely food insecure. This classification was based on the food insecurity score, a sum of scores from nine questions about food vulnerability or stress and coping behaviors with respect to experience and severity (frequency in the previous four weeks) [18]. Having a kitchen garden was recorded as ‘Yes’ when present and ‘No’ when it was not available. Wealth status, ordered in quintiles, from the richest (first quintile) to the poorest (fifth quintile).

Further, as a psychosocial variable, maternal social support was defined from six questions: having a friend or family member that (1) will assist the mother if she becomes ill, (2) will share food with her, (3) will share their house with her, (4) will lend her money, (5) will help her with guidance to improve her situation when she has problems, and (6) will offer support to her if she runs into personal problems. The answers were scored and the sum out of six and categorized as No support (for zero as score), low support (1 or 2 points), moderate support (3 or 4 points), and High support (5 or 6 points).

#### Statistical analysis

The outcome and explanatory variables were first described with frequencies and proportions. The wealth inequality in childhood stunting was quantified using the concentration index (CIX) and visually represented with the concentration curve [19].

The CIX measures the level of wealth-related inequality in a health outcome and is defined as twice the area between the concentration curve and the line of equality (the 45-degree line). It ranges from − 1 to + 1, where a negative CIX indicates that the health outcome (stunting in our study) is concentrated among the poor (pro-poor inequality), and a positive CIX indicates concentration among the rich (pro-rich inequality). The sign of the CIX reflects the position of the concentration curve relative to the line of equality. When the curve lies above the equality line, the CIX is negative, indicating pro-poor inequality; when it lies below the equality line, the CIX is positive, indicating pro-rich inequality. A CIX of zero would indicate no wealth inequality in stunting. The CIX is computed using the formula:

$$\:CIX=\:\frac{2}{\mu\:}\sum\:_{i-1}^{N}{y}_{i}{R}_{i}-1$$

Whereby *y*_*i*_ is the health variable of interest (stunting in this study); *µ* is the mean of *y*_*i*_; *R*_*i*_ is the fractional rank of individual i in the socio-economic distribution (in this study), i taking the value of 1 for the poorest and the value of n for the richest; and; *N* is the sample size.

To determine the contribution of the socio-economic variables to the observed inequality in childhood stunting, the CIX was decomposed following the Wagstaff approach [20]. This approach is based on a linear additive regression model, the CIX for child stunting (*y*) can be expressed as follows:

$$\:CIX=\sum\:k({\beta\:}_{k}{\stackrel{-}{x}}_{k}/\mu\:){C}_{k}+GC\epsilon\:/\mu\:$$

where *µ* is the mean of the outcome *y*, $\:{\stackrel{-}{x}}_{k}$ is the mean of the explanatory variable *x*_*k*_, *C*_*k*_ is the concentration index for *x*_*k*_, $\:{\beta\:}_{k}$​ is the regression coefficient for *x*_*k*_, and *GCε* is the generalized concentration index for the error term ε. The *C* is equal to a weighted sum of the concentration indices of the *k* determinants, where the weight for *x*_*k*_ $\:({\eta\:}_{k}={\beta\:}_{k}\frac{{\stackrel{-}{x}}_{k}}{\mu\:})$ is the elasticity of *y* with respect to each *x*_*k*_. The residual component, i.e., captured by the last term, reflects the wealth-related inequality in childhood stunting that is not explained by systematic variation in the regressors (covariates) by socio-economic status. As child stunting is a binary outcome, we applied a nonlinear extension of this decomposition approach, as the linear model assumptions do not hold. Specifically, we estimated a probit model and computed marginal effects to approximate the partial effects of each covariate. These marginal effects were then used in place of the linear coefficients $\:{\beta\:}_{k}$​ to compute the elasticities and derive the contributions of each determinant to the overall CIX. Finally, in line with previous applications of the method for binary outcomes, we normalized the CIX by dividing it by 1-*µ*; for consistent interpretation [20, 21].

The decomposition table reports the regression coefficient (βₖ), which represents the marginal effect (or partial effect in a probit model) of determinant xₖ on the probability of childhood stunting. Elasticity indicates the relative importance of a determinant, showing how sensitive the outcome is to a change in the explanatory variable while considering their means. It is calculated as the product of the coefficient and the mean of the determinant, divided by the mean of the outcome. The contribution shows the absolute contribution of each determinant to the total CIX (overall inequality). It is obtained by multiplying elasticity by the concentration index of the determinant. Percentage Contribution reflects the relative share of each determinant in explaining the total inequality, calculated as the contribution of a determinant divided by the total CIX. The residuals indicate the extent to which the inequality in childhood stunting remains unexplained, suggesting the potential impact of other, unmeasured variables. We analyzed our data using Stata version 18 [22].

#### Ethical considerations

Before data collection, ethical approval was obtained from the University of Rwanda College of Medicine and Health Sciences Institutional Review Board (IRB), with reference number 295/CMHS/IRB/2022. This approval is unique for a multidisciplinary study involving eight doctoral students and two postdoctoral fellows. All participants provided written informed consent to participate in the study. All information obtained from the respondents was kept confidential. Participation in the study was voluntary. The study participants were not required to answer any questions that made them feel uncomfortable, and they could withdraw from the study at any time without facing any negative consequences.

## Results

### Description of the study participants

Regarding demographic characteristics, the mean age of the 601 included children was 17.1 months, with the majority being girls (51.8%). On average, their mothers were aged 31.6 years, married (69.4%), and from the Gicumbi district (24%). Regarding socio-economic characteristics, most study participants were from severely food insecure households (43.7%), with kitchen gardens (74.5%), whose parents had less than a primary level of education (40.3% for mothers and 43.2% of fathers) and were either mothers’ partners who are engaged in non-skilled work or unemployed (93%). Regarding psychosocial characteristics, 61.2% of mothers reported having received social support during the two weeks preceding the survey. The prevalence of stunting was 27.1% in children aged 1–36 months (Table 1).

**Table 1:** Distribution of key demographic and socio-economic characteristics of the study population

Characteristics	Frequency	Percentage	
Demographic characteristics			
Child’s age (in months)			
Mean (SD)	17.1 (10.0)		
1–11 months	199	33.1	
12–23 months	213	35.4	
24–36 months	189	31.5	
***Child’s gender***			
Male	290	48.3	
Female	311	51.8	
***Mother’s age (in years)***			
Mean (SD)	31.6 (7.2)		
17–29 years	238	39.7	
30–39 years	269	44.8	
40–58 years	93	15.5	
***Marital status of the mother***			
Single	70	11.7	
Cohabitating	114	19.0	
Married	417	69.4	
***District of residence***			
Burera	129	21.5	
Gakenke	130	21.6	
Gicumbi	144	24.0	
Musanze	96	16.0	
Rulindo	102	17.0	
Socio-economic characteristics			
***Household’s wealth category (quintiles)***			
Richest (5th quintile)	108	18.7	
Richer	114	19.7	
Middle	120	20.8	
Poorer	113	19.6	
Poorest (1st quintile)	123	21.3	
***Household food insecurity Access***			
Food secure	74	12.4	
Mildly food insecure access	50	8.38	
Moderately food insecure access	212	35.5	
Severely food insecure access	261	43.7	
***Having a kitchen garden***			
Yes	448	74.5	
No	153	25.5	
***Mother’s education***			
Less than primary	219	40.3	
Primary level	193	35.5	
Secondary or higher	131	24.1	
***Father’s education***			
Less than primary	220	43.2	
Primary level	186	36.5	
Secondary or higher	103	20.2	
***Father’s main occupation***			
Employed	42	7.0	
Unemployed	559	93.0	
Psychosocial characteristics			
***Maternal social support***			
No support at all	149	25.1	
Low support	123	20.7	
Moderate support	150	25.3	
High support	171	28.8	
Nutrition status of the child			
No stunting	438	72.9	
Stunting	163	27.1	
Total	**601**	**100.0**	

### Wealth inequality in childhood stunting

**Fig. 1:**
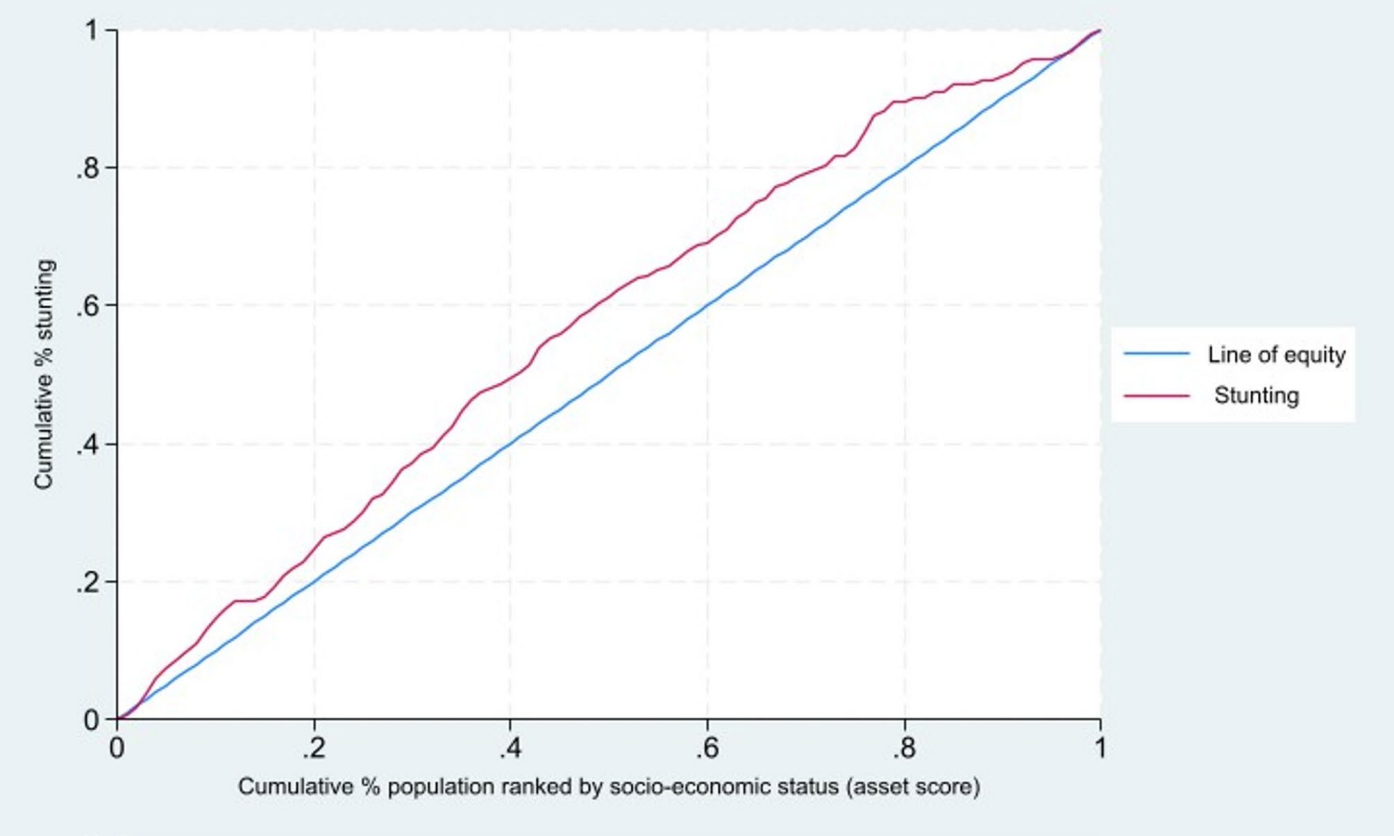
Concentration curve of wealth inequality in childhood stunting in the Northern province, Rwanda

The concentration curve lies above the line of equality (the 45-degree line), showing that stunting is more concentrated among poorer households (Fig. 1). This visual representation is consistent with the calculated overall negative concentration index (CIX = − 0.19; 95% CI: − 0.30 to − 0.08). The negative value confirms pro-poor inequality, meaning that children from the worse-off households are disproportionately affected and bear a greater burden of stunting compared to their better-off counterparts. The area between the curve and the line of equality shows the extent of wealth-related disparity.

### Decomposition of the concentration index

The results of the decomposition analysis are presented in Table 2. The selected determinants accounted for 165.2% of the inequalities, with residuals of −65.2%, indicating an offset effect on the overall contribution or share of inequality unexplained by the model. The analysis revealed that socio-economic determinants contributed the highest to the overall CIX, accounting for 147.9% of the inequalities; namely wealth index (70.7%). The poorest quintile contributing − 0.155, or 81.6%, of the total CIX indicates its disproportionately considerable influence on stunting inequality, while the richer quintile contributed positively (0.076 or − 39.9%), slightly offsetting the concentration of stunting among the poorest. Partner’s occupation was another major contributor with 42.8%, namely the unemployment or non-skilled occupation status. Food insecurity (34%), whereby severely food insecure households contributed − 0.099 (52.3%) to inequality, while mild food insecure ones slightly offset the concentration, contributing 0.025 or −13.4%.

The demographic determinants contributed in the second order, especially Musanze district (8.4%), children aged 12–23 months (5.3%), and males (4.4%). Psychosocial determinants followed, namely maternal social support (7.2%).

**Table 2:** Summary of the results of decomposition analysis for childhood stunting inequalities in Northern province, Rwanda

Variable	Coeff.	Cix	Elast.	Contr.	% Contr.
Demographic determinants					
Child’s age (in months)					
0–11 months	Ref.				
12–23 months	**0.286**	−0.027	0.374	−0.010	5.3
24–36 months	**0.419**	0.019	0.486	0.009	−4.9
Child’s gender					
Females	Ref.				
Males	**−0.089**	0.049	−0.169	−0.008	4.4
Maternal age in years					
17–25	Ref.				
26–35	−0.004	0.023	−0.007	0.000	0.1
36 and above	−0.010	0.114	−0.012	−0.001	0.7
Marital status of the mother					
Single	−0.006	−0.217	−0.002	0.001	−0.3
Cohabitating	−0.023	−0.309	−0.016	0.005	−2.7
Married	Ref.				
District of residence					
Rulindo	Ref.				
Gicumbi	0.068	0.113	0.060	0.007	−3.6
Gakenke	0.035	−0.122	0.028	−0.003	1.8
Burera	0.035	−0.051	0.028	−0.001	0.7
Musanze	**0.172**	−0.158	0.101	−0.016	8.4
***Sum***					***10***
Socioeconomic determinants					
Maternal education					
Illiterate	**−0.111**	−0.054	−0.165	0.009	−4.7
Primary	−0.064	−0.001	−0.083	8.3E-05	0.0
Secondary or higher	Ref.				
Paternal education					
Illiterate	0.007	−0.116	0.011	−0.001	0.7
Primary	0.009	0.022	0.013	0.000	−0.1
Secondary or higher	Ref.				
Partner’s main occupation					
Employed	Ref.				
Unemloyed	0.066	−0.362	0.225	−0.081	42.8
Household wealth quintile					
Richest	Ref.				
Richer	**0.194**	0.535	0.141	0.076	−39.9
Middle	**0.145**	0.031	0.111	0.003	−1.8
Poorer	**0.172**	−0.472	0.124	−0.058	30.8
Poorest	**0.197**	−1.002	0.155	−0.155	81.6
Household food insecurity category					
Food secure	Ref.				
Mildly food insecure	**0.226**	0.4	0.070	0.025	−13.4
Moderately food insecure	0.122	0.1	0.2	0.009	−4.9
Severely food insecure	**0.183**	−0.3	0.295	−0.099	52.3
Having a kitchen garden					
Yes	Ref.				
No	0.030	−0.3	0.028	−0.009	4.7
***Sum***					***147.9***
Psychosocial determinants					
Maternal social support					
Not support at all	0.039	−0.4	0.036	−0.014	7.1
Low support	0.045	0.1	0.034	0.003	−1.4
Moderate support	−0.055	0.1	−0.052	−0.003	1.6
High support	Ref.				
*** Sum***					***7.2***
*** Sum***				**−0.313**	**165.2**
*** Concentration index (CIX)***				**−0.190**	
*** Standard error (s.e)***				**0.054**	
*** Residual***				**0.124**	**−65.2**

*Bolded coeff*. indicates p < = 0.05; *Coeff***.**: Coefficient from the probit model regression for Stunting; *Cix*: Determinant Concentration index; *Elast***.**: Elasticity; *Contr*: Contribution to concentration determinant index; **% ***Cont*r: Percentage contribution to the overall *CIX*; *Ref*.: Reference category of the variable

## Discussion

This study aimed to estimate the wealth-related inequalities of childhood stunting in the Northern Province of Rwanda and to identify the contributors to those inequalities. We found that childhood stunting was concentrated among the poor, with household wealth index, partner’s occupation, household food insecurity, living in Musanze district, and maternal social support, as the main contributors to the inequality. The concentration index was − 0.190. While this value falls within the range reported in other low- and middle-income country studies, direct comparisons must be interpreted with caution because most published estimates—such as those from Nigeria [23], Tanzania [24], Ethiopia [25], Zimbabwe [26], and Nepal [27]—are based on nationally representative RDHS datasets, whereas our estimate is derived from a subnational sample, one region of the country. Differences in population coverage, sampling design, and regional heterogeneity may account for part of the observed variation. The added value of our subnational analysis lies in its ability to uncover within-country disparities that national averages may obscure. In the Northern Province context—characterized by higher poverty prevalence and a heavier stunting burden than other provinces, the relatively higher negative CIX reveals a substantial pro-poor concentration of stunting. While not directly comparable national CIX for stunting in Rwanda has been published, our findings are broadly consistent in direction with global evidence showing that stunting disproportionately affects poorer households [25, 28]. However, the magnitude observed here underscores the depth of inequities in this province and signals the need for geographically targeted interventions.

Our finding that wealth was the main socio-economic contributor to the observed inequalities in childhood stunting contributes to the large body of evidence from resource-limited settings, showing consequential disparities between the richest and poorest households on stunting [23–25, 27, 29–31]. Similarly, higher wealth contributions are reported in studies from Tanzania, Ethiopia, Zimbabwe, and Nepal [24–27], and lower in Bangladesh [32]. Households living in poverty expose their members to a multitude of hardships in striving for survival; inadequate nutrition, i.e., restricted access to diverse and nutritious foods, as well as food insecurity, lack of information about and time to apply beneficial child care practices, lower access to health care and higher risk of infections [10, 33]. In Rwanda, 24,900 cases of stunting are attributable to childhood diarrhoea, making it a disease of the poor [7], as children from poor families are more likely to be exposed to pathogenic agents and suffer from environmental enteropathy [34], which is further exacerbated by low access to health services [35]. It is also reported that 45.3% of the population of the Northern Province are classified as poor, a higher proportion of households struggling for survival, with a very negative impact on children’s growth, thus stunting [3]. Therefore, household financial support programs targeting poor communities and the most vulnerable could reduce wealth inequalities and related child stunting rates [23, 36, 37].

The second most significant contributor to wealth-related inequalities in childhood stunting was the father’s engagement in non-skilled work or unemployment. To our knowledge, no study has ever reported the father’s main occupation as a contributor to the socio-economic inequalities in childhood stunting, whereas maternal factors are reported in Ethiopia and Tanzania [24, 25] and Rwanda [10]. In Rwanda, fathers are typically regarded as the primary providers for their families (gatekeepers), and their occupation greatly influences household wealth. Consequently, reliance on subsistence activities such as agriculture or animal husbandry—rather than on business or informal employment—has been associated with higher rates of stunting [31, 38–40]. Given that a large proportion of Rwandans are manual labourers, including agricultural workers, there is a pressing need to improve their economic conditions [3]. Therefore, having better conditions or salaries in no-skilled jobs would increase the household’s purchasing power to procure quality food and thereby meet the daily nutritional needs of children [41, 42]. Policies like revisiting wage policies (in view of the very low minimum wage in Rwanda) and job training for low-skilled population are encouraged.

The third most significant contributor to wealth-related inequalities was household food insecurity. This finding is consistent with a study from Zimbabwe, which reported that household food security is disproportionately concentrated among the wealthy, but to higher extent than this study [26]. Poor households often struggle to provide their children with food rich in bioavailable zinc and iron, such as meat, poultry, fish, or eggs, compared to children from better-off families [35]. Many households feel rather interested in more voluminous food for the entire family, which is, in most cases, less nutritious [43]. In Gicumbi, one of the five districts of the Northern Province, higher odds of severe stunting were reported in children from households with moderate and severe food insecurity [44]. Additionally, at the national level, 10.2% of the overall inequality in stunting is attributed to household food insecurity [10]. As low income remains a major driver of household food insecurity [45]; food system policies targeting the most vulnerable could have the potential to bridge the wealth disparities associated with higher stunting rates in food-insecure communities [37], including the Northern Province. Such policies could include food subsidy and targeted food baskets.

The fourth contributor to wealth-related inequalities in childhood stunting was living in Musanze district, as a considerable demographic determinant, contributes 8.4%. Contrarily, a few studies have found very low or no regional or subnational contribution to the socio-economic inequalities in childhood stunting [32, 46, 47]. Stunting is proven to be clustered in the Northern province, with hotspots in Musanze, Gakenke and Gicumbi districts [48]. More specifically, Musanze is one of the districts with higher stunting rates than the national average [49]. Interestingly, a study has found that farming as the main source of income is negatively associated with stunting in Musanze district [50]. With Musanze being one of the most populated districts in Rwanda, the competition for arable land is high. Interventions aimed at improving the well-being of households in Musanze, such as economically sustainable alternatives to agriculture, could help decrease wealth inequalities and stunting.

A key novelty of this study is the inclusion of psychosocial determinants, particularly maternal social support, in the decomposition of wealth-related inequalities in childhood stunting. In our analysis, maternal social support accounted for 7.2% of the total wealth inequality, underscoring its relevance alongside more traditionally examined socio-economic and demographic factors. A different analysis work that used the same data found a higher risk of stunting in the absence of support during the mother’s illness and lack of guidance during problem-solving [33]. Evidence from other contexts aligns with this, showing that mothers with higher social support are more likely to effectively feed their children [51]. For instance, mothers’ ability to identify anyone to borrow food or money improves the feeding and nutritional status of their children. On the other hand, psychosocial stress and isolation may impair caregiving practices [52]. The integration of psychosocial measures into equity analysis broadens the scope of determinants considered but also highlights actionable policy entry points. As a key insight, social support interventions such as peer support networks, community-based parenting groups, microfinance schemes, and cash-plus interventions could help reduce wealth-related inequalities in stunting.

### Strengths and limitations

This study has a few strengths. It consisted of a good sample size, which ensures the external validity of its findings. It utilizes data from a multistage cluster sampling study, creating robustness and power to provide accurate estimates. The use of decomposition stands out as a strong and complex method for introducing new knowledge to address stunting in Rwanda, particularly in estimating and explaining health inequalities. Nevertheless, the present study has several limitations.

A major limitation is the cross-sectional design, which hinders causal inference. It did not include information on some relevant socio-economic variables like rural-urban settings, which could have also contributed to the inequality. There could be a possible response bias among participants, as well as errors in anthropometric measurements by enumerators. However, we recruited nurses with research experience and emphasis was put on collecting as accurate data as possible, using well-calibrated tools (height boards and weight scales). Future studies should encompass a broader range of factors to inform more comprehensive interventions. More specifically, a comprehensive qualitative study around all identified determinants could shade more lights towards interventions.

## Conclusion

The study found that stunting was concentrated among the poor. The decomposition of the concentration index revealed that socio-economic factors, namely the wealth index, partner’s occupation, and household food insecurity, contributed most to explaining the wealth inequalities in childhood stunting. Next came Musanze district as a demographic factor, and maternal social support as a psychosocial factor. Demographic factors contributed the least to the overall wealth inequalities.

The findings of this study underscore the need for targeted equitable interventions to promote economic empowerment among poor households, ensuring household food security, having better conditions/salaries in no-skilled jobs, more focused interventions in Musanze district, and providing social support with mothers facing challenges within these households.

## Data Availability

The datasets used and/or analysed during the current study are available upon request to the project coordinator at [https://www.gu.se/om-universitetet/hitta-person/gunillakrantz2](https:/www.gu.se/om-universitetet/hitta-person/gunillakrantz2).

## Abbreviations

CIX: Concentration Index
CMHS: College of Medicine and Health Sciences
HAZ: Height-for-Age z-score
IRB: Institutional Review Board
LMICs: Low- and middle-income countries
PCA: Principal component analysis
RDHS: Rwanda Demographic and Health Survey
SD: Standard deviation
UNICEF: United Nations Children’s Fund
WHO: World Health Organization
